# Clinical evaluation and management of badminton-related eye injuries: a retrospective case series

**DOI:** 10.1186/s12886-023-02972-8

**Published:** 2023-06-07

**Authors:** Tingting Guo, Wanru Shi, Xiuqian Yi, Tianrui Huang, Peijie Huang, Kang Xue

**Affiliations:** 1 Department of Ophthalmology and Shanghai Key Laboratory of Visual Impairment and Restoration, Eye, Ear, Nose, and Throat Hospital of Fudan University, Shanghai, China; 2 Shanghai West Yanan Middle School, Shanghai, China; 3 Department of Critical Care Medicine, School of Medicine, Shanghai General Hospital, Shanghai Jiaotong University, Shanghai, China

**Keywords:** Eye injury, Ocular trauma, Badminton-related, Ocular trauma score, Presenting visual acuity, Final visual acuity

## Abstract

**Background:**

To describe the clinical features, visual outcomes, management, and complications of ocular injury in badminton and investigate risk factors associated with visual impairment.

**Methods:**

Data on patients injured while playing badminton admitted to Department of Ophthalmology, Eye, Ear, Nose, and Throat Hospital, Fudan University between January 2018 to December 2020.The relationship between visual acuity (VA) and demographic and clinical variables was also analyzed. Patients were managed medically or surgically as per their needs, followed up for at least 18 months. The visual outcomes were predicted using ocular trauma score (OTS), predicted outcomes were compared with actual outcomes using statistical tests.

**Results:**

This study involved 102 patients (78 men, 24 women) with a mean age of 43.8 ± 16.1 years (7–71 years). Of these, 93 patients had closed-globe injuries and 9 had open-globe injuries. Vision-threatening findings included lens subluxation(31.4%),retinal detachment(13.7%),hyphema(12.7%). Open-globe injury had significantly lower presenting VA and final VA (*P*= 0.0164, 0.0053).Final VA was found to be correlated with presenting VA, maculopathy, retinal detachment, and OTS (*P*=0.0000, 0.0494, 0.0001, 0.0000 respectively), it was worse in patients who were under 20 years of age and were female. OTS prediction was not significantly different when compared with actual visual outcomes postoperatively in OTS3, OTS4, and OTS5 (*P* > 0.05),while the prognosis of patients with OTS1 and OTS2 was better than OTS study (*P*=0.001, 0.007, respectively).

**Conclusion:**

Badminton-related closed-globe injuries were more frequent; open-globe injuries were usually more serious. Younger and female patients have poorer visual recovery prognoses. OTS was found to be a reliable tool for predicting visual outcomes.

## Introduction

The popularity of badminton is rapidly increasing in China, leading to an increased incidence of physical and eye injuries. Although eye injuries rarely occur, they can lead to devastating and disabling consequences. Worldwide, approximately 1.6 million people become blind from traumatic eye injuries, and approximately 2.3 million people with bilateral low vision suffer from ocular trauma every year [1]. Thus, evaluating trends in incidences of injuries related to badminton is important. The ocular trauma score (OTS) is a model that has been proposed for predicting visual outcomes based on an initial examination [2, 3]. Although this model has proved to be effective in the common ocular injuries of general population, few studies have tried to validate prognostic models in cases of badminton-related eye injuries.

A total of 102 patients who had acquired eye injuries when playing badminton and who presented to the outpatient and emergency departments were enrolled in this study. This study aimed to identify the characteristics of badminton-related eye injuries and analyze the risk factors associated with visual impairment at a patient’s first hospital visit to emphasize the importance of awareness regarding badminton-related eye injuries and provide suggestions for appropriate ocular protection in badminton.

## Methods

This was a retrospective case series study, it included the review of medical histories of all patients with badminton-related eye injuries admitted to the Department of Ophthalmology, Eye, Ear, Nose, and Throat Hospital, Fudan University, Shanghai, China, from January 2018 to December 2020. There were102 such cases in total. Data, including age, sex, presenting visual acuity (VA), time of presentation, duration of decreased vision, type of intervention (medical or surgical), sequelae, complications, and final visual acuity were collected on a standardized form. A detailed history of every patient was taken at his/her first hospital visit. Examination of the anterior segment was performed by slit lamp, intraocular pressure(IOP) was recorded by noncontact tonometer, and the posterior segment was examined in slit lamp with + 90D lens lamp. Surgically managed patients were examined on postoperative day 1, day 7, day 15, and day 30, and further, they were examined after the intervals of 6 months,12 months and 18 months. The classification of eye injuries was based on the Birmingham Eye Trauma Terminology (BETT) and the Ocular Trauma Classification System (OTCS). In brief, six predictive factors: VA, rupture, endophthalmitis, perforating injury, retinal detachment, and relative afferent pupillary defect (RAPD) at the initial visit were summarized and calculated. Then the patients were classified as OTS categories (1 to 5).

The presenting VA was categorized into three categories: poor vision (VA < 6/60),moderate vision (VA ≥ 6/60– ≤6/18), and good vision (VA > 6/18). The raw scores of the patients were calculated according to Table 1 and the obtained raw scores were converted to OTS according to Table 2.The study was conducted according to the guidelines of the Declaration of Helsinki, and the study protocol was approved by the institutional review board of the Eye, Ear, Nose, and Throat Hospital at Fudan University.

**Table 1:** Calculation of OTS, variables, and raw score

Variables	Raw score
A,Initialvision	
No PL	60
PL/HM	70
1/200–19/200	80
20/200–20/50	90
≥ 20/40	100
B,Rupture	**-23**
C,Endophthalmitis	**-17**
D,Perforating injury	**-14**
E,Retinal detachment	**-11**
F,Afferent pupillary defect	**-10**

**Table 2:** Comparison of ocular trauma score–predicted vision with actual vision

	No PL		HM to PL		1/200–20/190		20/200–20/50		≥20/40			
OTS type	*Achieved*	*OTS*	*Achieved*	*OTS*	*Achieved*	*OTS*	*Achieved*	*OTS*	*Achieved*	*OTS*	*χ* ^2^	*P-value*
OTS1	0	73	71.4	17	14.3	7	14.3	2	0	1	18.5924	0.001
OTS2	0	28	6.3	26	43.8	18	31.3	13	18.8	15	14.1206	0.007
OTS3	0	2	16.7	11	26.7	15	30	28	26.7	44	4.7432	0.315
OTS4	0	1	4	2	0	2	36	21	60	74	3.5089	0.477
OTS5	0	0	0	1	0	2	12.5	5	87.5	92	2.4505	0.484

HM: hand movement, OTS: ocular trauma score, PL: perception flight

Analyses of the data were conducted using SPSS 18.0 software. The data are presented as the mean value with the standard deviation. The categorical variables final VA and OTS were compared with the χ^2^ or Fisher’s exact test.The degree of correlation and statistical significance between variables were analyzed using Pearson’s and Spearman’s rank correlation tests for normally and nonnormally distributed data, respectively. A *P-*value of less than 0.05 was considered statistically significant.

## Results

The study included 102 eyes of 102 patients between 7 and 71 years of age (43.8 ± 16.1 years), the median age was 46 years. Patients 20 years of age or younger were 11(10.8%), whereas patients older than 20 years of age were 91(89.2%). Of the 102 patients, 78 (76.5%) were male and 24(23.5%) were female; 46 (45.1%) patients had an injury to the right eye and 56 (54.9%) to the left eye; 93(91.2%) patients had closed-globe injuries and 9 (8.8%) had open-globe injuries. Professional training or safety education was received by 9 (8.8%) instigators. 80 (78.4%)patients were hit by shuttlecock, while 22(21.6%) patients were by racket;63(61.8%) patients were actively engaged in the game players who were hit by their partner, 37(36.3%) patients were hit by opponent,2 (2.0%)patients were hit as a bystander, none of the patients wore protective eyewear when they played badminton. None of badminton player hit themselves, regarding refractive error correction, 20(19.6%) patients were wearing eyeglasses, four of 20 patients suffered from broken glasses, none of all patients had undergone refractive surgery. All the patients with penetrating eye injuries in this study suffered extremely serious vision impairment. Five of nine open-globe injuries cases were children below 12 years of age. Four of the nine open-globe injuries resulted from shattered eyeglasses, the shuttlecock hit the glasses, which broke and punctured his or her eyeball; three patients were struck by an opponent’s shuttlecock, two patients had their eyes broken by a partner’s badminton racket. Open-globe injury cases had significantly lower presenting and final VA(P=0.0164, 0.0053, respectively).

**Fig. 1:**
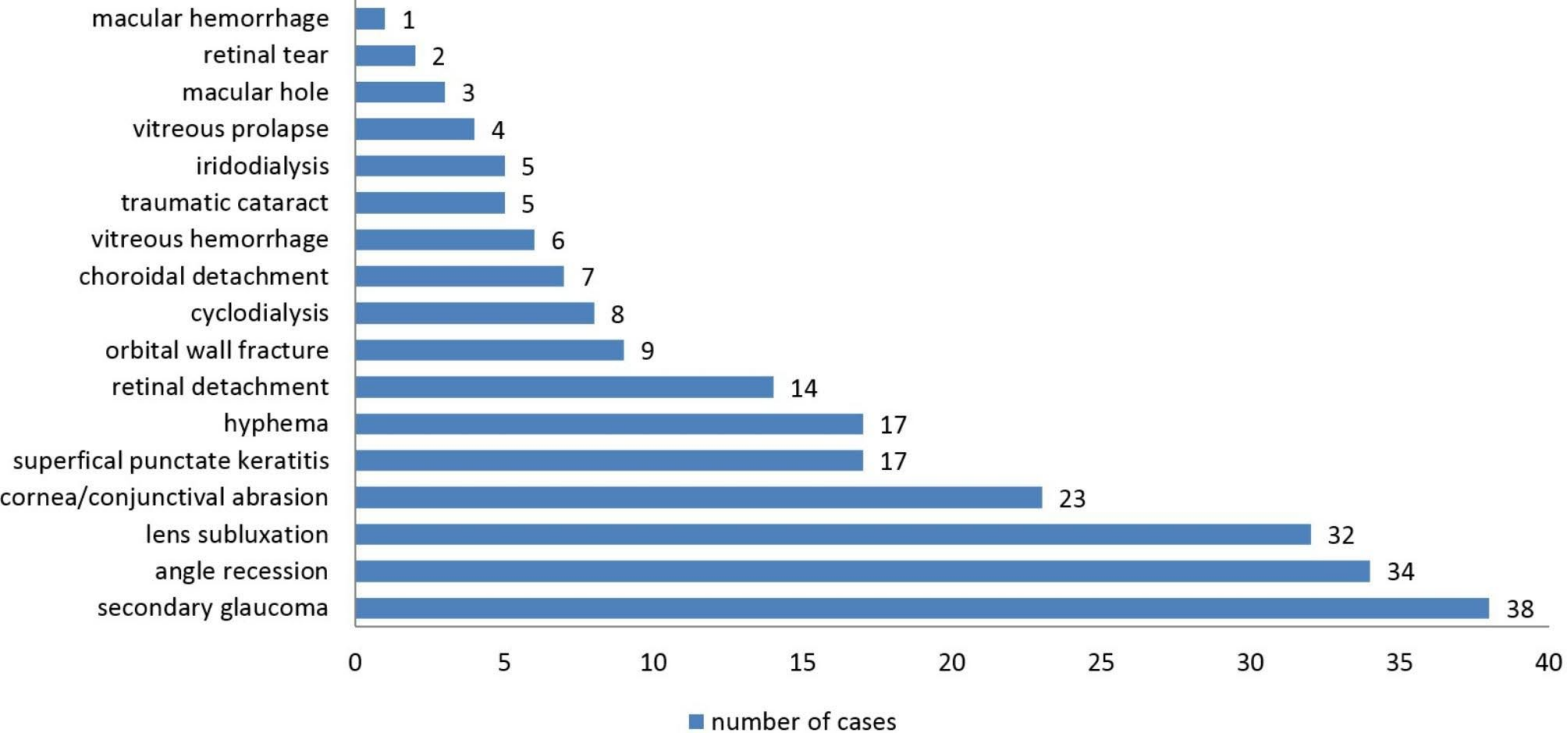
Distribution of diagnosis of badminton-related eye injury at first hospital visit

**Fig. 2:**
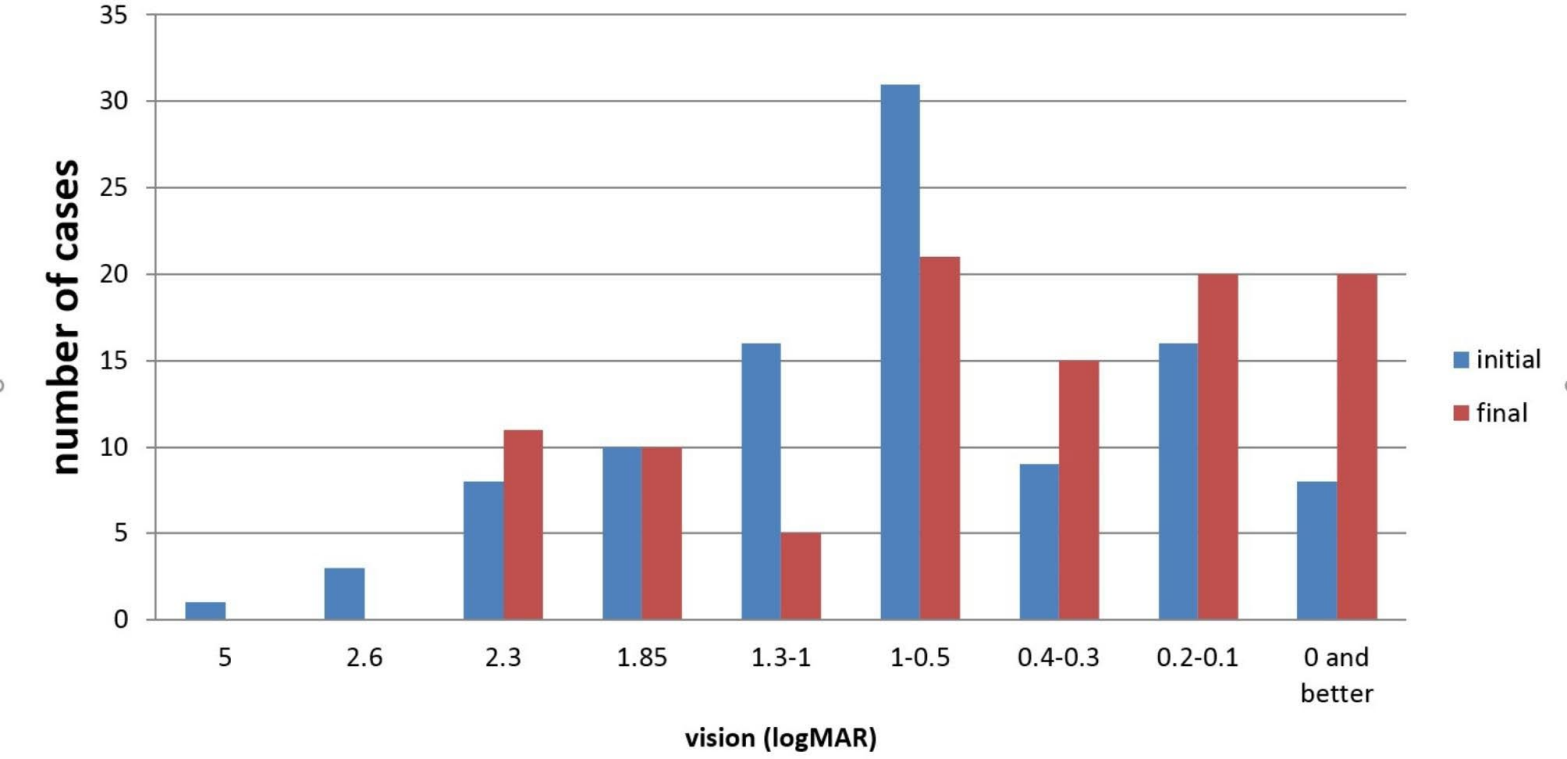
Vision outcomes at the initial and final visits

Cases presented with a variety of clinical findings, including diminution of vision. The most frequent eye injury diagnostic categories were secondary glaucoma(37.3%),angle recession (33.3%), and lens subluxation (31.4%).Vision-threatening findings in the decreasing order of frequency were lens subluxation(31.4%),retinal detachment(13.7%), hyphema (16.7%), intravitreous hemorrhage(5.9%), and traumatic cataract (4.9%). The mean IOP at presentation was 22.6 mmHg. In this study, 31 (30.4%)cases had high IOP at the first presentation, and 19(18.6%) cases had secondary closed-angle with ultrasound biomicroscopy examination. Presenting VA was found to have a significant positive correlation with final VA(*P*=0.000),indicating that good initial vision results in better final VA(Table 3). At their first visit to the hospital, 70(68.6%)patients complained of visual impairment. The diagnosis categories of 102 patients are shown in Fig. 1. The initial and final VA data are presented in Fig. 2.

**Table 3:** Presenting VA has a significant positive correlation with final VA

Presenting VA	Final VA			Total, *n*(%)	*χ*^2^, *P-value*
	Poor vision, *n*(%)	Moderate vision, *n*(%)	Good vision, *n*(%)		
Poor vision, *n* (%)	24(92.3%)	8(38.1%)	6(10.9%)	38(37.3%)	
Moderate vision, *n* (%)	1(3.8%)	11(52.4%)	19(34.5%)	31(30.4%)	
Good vision, *n* (%)	1(3.8%)	2(9.5%)	30(54.5%)	33(32.4%)	**χ2**=59.37
**Total**, ***n*(%)**	26(100%)	21(100%)	55(100%)	102(100%)	*P*=0.000

The analysis revealed that the male and female patients had no significantly different distributions of diagnoses. Both genders had similar presenting VA, but sex-specific differences in final VA were noted, where females seemed to have poorer final VA (*P*=0.025).The final VA was worse in patients under 20 years of age(*P*=0.0254). Importantly, the final VA was found to have a correlation with the initial VA(*P*=0.0000,*R*=0.7100), maculopathy(*P*= 0.0494,*R*= 0.690),retinal detachment (*P*=0.0001,*R*= 0.540), and OTS (*P*=0.0000, *R*=−0.7076).The final VA distribution of the cases in this study and the OTS study with respect to the OTS categories are shown in Table 2.No significant difference was found for patients with OTS3, OTS4,or OTS5 (*P*=0.315,0.477,0.484, respectively);however, there were significant differences for patients with OTS1 and OTS2 (*P*=0.001, 0.007, respectively).Out of 102 cases, 75 (73.5%) required surgical intervention (Table 4).

**Table 4:** Details of surgical intervention of 75 cases

Surgical approaches	*n*	(%)
**Glaucoma Surgical Therapy**	29	28.4
Laser peripheral iridology	3	2.9
Anterior chamber irrigation	3	2.9
Express drainage device implantation	7	6.9
Pars plana drainage implantation	4	3.9
Trabeculectomy	7	6.9
Microsuture of ciliary body	5	4.9
**Lens Surgery**	**28**	**27.5**
P + AV	2	2.0
P + transsclerally sutured IOL	10	9.8
P + I + MCTR + AV	5	4.9
**Vitreoretinal Surgery**	**28**	**27.5**
Endolaser	2	2.0
Suture + intraocular injection	9	8.8
Cryotherapy + scleral buckle	3	2.9
PPV + SOI + endolaser	14	13.7

MCTR: modified capsular tension ring, P: phacoemulsification, IOL: intraocular lens, SOI: silicon oil injection, AV: anterior vitrectomy, PPV: pars plana vitrectomy

## Discussion

Sports-related eye injuries are not uncommon; however, sports activities at a particular geographical location depend on the socioeconomic status and environment of that region [4–7]. Although ocular trauma due to badminton is a less-reported incident in the literature, it is found to be growing in Shanghai. Thus, it is necessary to analyze the clinical characteristics and risk factors for eye injuries on the basis of region of incidence to prepare a specific regional prevention program for sports-related eye injuries.

Notably, eye injuries were specifically explored in this study, and visual sequelae secondary to trauma were examined. The proportion of patients who complained of ocular injuries at their first hospital visit was consistent with similar studies [8–10]. In this study, secondary glaucoma, angle recession, lens subluxation, contusion/abrasion were the most prevalent diagnoses, and contact with the shuttlecock was the most common cause of eye injury. Short-term or long-term visual sequelae were reported in 68.6% of the patients, emphasizing the importance of recognizing potential complications of badminton-related eye injury. Closed-globe injuries were found to be less severe than open-globe injuries. Elevated IOP after traumatic angle damage is generally due to angle recession and lens subluxation, which is associated with short-and long-term sight-threatening risks [11, 12]. Yu et al. reported that hyphema is the most common injury, and 49.4% of patients experienced angle recession [3]. Lee et al. found that 26.1% of patients experienced contusion/abrasion after an injury [8]. Proportion of angle recession and contusion/abrasion in our case series was 33.3% and 22.5% respectively.In the study, 19 patients (18.6%) experienced secondary angle on UBM, as for the reason, the proportion of lens subluxation in this group exceeded 30%,which may lead to lens dislocation related angle closure. Another reason may due to inflammation-related synechiae, as inflammation is one of the major reactions from trauma.

The most common mechanism of injury is blunt trauma to the eye [13, 14]. In most cases, open-globe injuries are less common but usually more serious than closed-globe injuries because they require surgical intervention and result in poorer vision outcomes [15]. Most of those injured were unaware of the high-risk the nature of badminton poses for ocular injuries. The cases of open-globe injury had significantly poorer presenting and final VA. There were nine patients with open-globe injuries in this study, these nine patients suffered extremely serious vision impairment, four of nine injuries resulted from shattered eyeglasses. The use of spectacles is also associated with increased frequency and severity of injury because of the risk of penetrating ocular injury caused by shattered glasses; thus, spectacles should not be worn by badminton players [10, 16]. Players requiring spectacle correction can wear contact lenses,prescription protectors complying with the standards, or an eye-shield style protector over their own spectacles [14, 17] . The other five cases of eyeball rupture were juveniles without eyeglasses, the eyeball structure of whose was perhaps not as strong as that of adults [18] .

Noble et al. evaluated that injury proportions were higher in males and those in their 10s to early 20s in age distribution [19]. In this study, the proportion of individuals in their 50 and 60 s was 28.4% and 12.7%, respectively. However, the final VA was worse in female patients and those under 20 years of age. Pediatric ocular trauma has a poor prognosis; hence, it is a burden to society. It is in Sweden that it became mandatory for children under 15 years of age to wear eye protection during floor sport activities, and the risk of eye injury was reduced to minimum [20]. To prevent eye injuries, restriction of participation is worth recommending in badminton for certain populations who are at higher risk of ocular injuries. The data in this study are consistent with previous studies that have shown initial VA to be the most predictable risk factor for postoperative outcomes [6, 8, 10]. Badminton generally results in a few eye injuries, but serious complications, such as retinal detachment, vitreous hemorrhage, macular hole, retinal tear, and macular hemorrhage can occur [8, 10]. In this study, post traumatic retinal detachment and vitreous hemorrhage rates of 13.7% and 5.9% respectively were noted. Traumatic maculopathy and retinal detachment have been confirmed as important contributors to poorer vision outcomes. In this study, the final VA was found to be correlated with maculopathy and retinal detachment. High intraocular pressure and hyphema may be transient, and traumatic cataract can be cured by surgery. The blurred vision caused by maculopathy and retinal detachment is usually irreversible.

The most widely used predictive model for ocular trauma is validated OTS; it has been reported that the prognostic outcome of OTS has been mostly accurate in several studies [2, 3]. In the current study, the OTS1 and OTS2 categories showed significantly better visual outcomes than the OTS study, whereas the prognosis for the other categories was in harmony with the OTS study and in accordance with the findings of Ocal’s [21]. For this reason, the number of cases for the OTS1 and OTS2 categories may be lower in this study. This paper proposes that evolving technology in recent years has made it possible to handle these serious cases, resulting in a better prognosis.

This study was not without certain limitations. First, the number of open-globe injury cases was limited. Second, all data were due to the retrospective nature of the analysis of this paper, which contained some unrecorded data.

## Conclusion

In this study, the most common diagnosis after injury were secondary glaucoma, angle recession, and lens subluxation in the order of frequency.The most common findings causing poor final visual outcomes were retinal detachment and maculopathy. Closed-globe of badminton-related eye injuries were more frequent, and open-globe injuries were usually more serious. Younger patients and females had poorer visual recovery prognoses. The OTS was found to be a reliable tool for predicting visual outcomes. However, more emphasis should be placed on preventive aspects, such as the use of protective eyewear, professional guidance, and safety education.

## Data Availability

All the data used to support the findings of this study are included within the article and are available from the corresponding author upon reasonable request.

## Abbreviations

OTS: Ocular trauma score
BETT: Birmingham Eye Trauma Terminology
VA: Visual acuity
IOP: Intraocular pressure
OTCS: Ocular Trauma Classification System .
